# Childhood-onset rheumatoid arthritis at a tertiary hospital in Senegal, West Africa

**DOI:** 10.1186/s12969-023-00889-6

**Published:** 2023-09-12

**Authors:** Mounib M. Sabounji, Hilaire Lissimo, Amina Deme

**Affiliations:** 1 Department of Rheumatology, Aristide Le Dantec Hospital, Dakar, Senegal; 2 Department of Rheumatology, COUD Hospital, UCAD, Dakar, Senegal; 3 Department of Paediatrics, UCAD, Dakar, Senegal

**Keywords:** Childhood-onset, Rheumatoid arthritis, Disease activity, Functional disability, Senegal

## Abstract

**Background:**

Childhood-onset rheumatoid arthritis (CORA), known as rheumatoid factor (RF)-positive juvenile idiopathic arthritis is a type of juvenile idiopathic arthritis that shares the same genetic factors and clinical features as adult-onset rheumatoid arthritis. In Africa, CORA hasn’t been the subject of a specific study.

**Objectives:**

The aim of this study is to describe the clinical features, disease activity, functional disability, and treatment of CORA at diagnosis in Senegal and compare the findings to other CORA populations.

**Methods:**

We conducted a mixed cohort study by reviewing the medical records of patients diagnosed with CORA with an age of symptom onset < 18 years according to the 2019 PRINTO provisional criteria for RF-positive JIA from January 2020 to December 2022 at rheumatology department of Aristide Le Dantec Hospital in Dakar, Senegal. We collected demographic, clinical, paraclinical and therapeutic data. Disease activity score was assessed by DAS28-ESR and DAS28-CRP. Functional disability was assessed using Health Assessment Questionnaire (HAQ) or Childhood HAQ.

**Results:**

A total of 21 patients were included. Eighteen (85.7%) were Females. The mean age at symptom onset was 13.0 ± 3.0 years, and at diagnosis was 16.4 ± 4.2 years. Morning stiffness, joint swelling, and joint deformities were found in 20, 18 and 13 patients respectively. Four patients had a family history of rheumatoid arthritis. Five patients had extra-articular involvement such as rheumatoid nodules. Two patients had interstitial lung disease. The biological inflammatory syndrome was found in 90% of cases. 16 of 21 (76.2%) patients had positive RF, and 18 of 20 (90%) patients had positive Anti-CCP. Seven of 12 (58.3%) patients had positive anti-nuclear antibodies. The mean DAS28-ESR was 5.7 ± 1.0. Fifteen (71.4%) patients had high disease activity (DAS28-ESR > 5.1). The mean DAS28-CRP was 5.4 ± 1.1. The median HAQ was 2.12 with a mean HAQ of 1.9. Nineteen (90.5%) patients were treated with methotrexate, while 17 (81%) had a combination of methotrexate and hydroxychloroquine. Oral prednisone was used in 17 (81%) cases. Non-steroidal anti-inflammatory drugs were used in 4 cases (19%). After 6 months of treatment, mean DAS28-CRP was 2.9.

**Conclusion:**

In our study, CORA mainly affects 13-year-old girls, characterised by high disease activity with joint deformity and significant functional impairment. Treatment is mainly based on methotrexate, prednisone and hydroxychloroquine. Further studies are needed to determine the exact clinical phenotype of this disease.

## Introduction

Rheumatoid arthritis (RA) is a chronic inflammatory systemic autoimmune disease that primarily targets synovial joints [1], although it can occur at any age, the peak incidence is at the age of 30–50 years [2–5]. It’s mainly characterized by chronic symmetrical polyarthritis involving hand and foot joints [6], extra-articular manifestations (encompass involvement of the skin, eye, heart, lung, renal, nervous, and gastrointestinal systems) [7] and positivity of rheumatoid factor and/or anti-cyclic citrullinated peptides antibodies (Ac-CCP) [8] which generally leads to progressive joint destruction, deformities and consequent disability [6, 9]. Children with positive RF and/or Ac-CCP polyarticular juvenile idiopathic arthritis share the same clinical features and genetic risk factors as adult-onset RA, representing childhood-onset rheumatoid arthritis (CORA) [10, 11].

In sub-Saharan Africa, Adult-onset rheumatoid arthritis has been previously studied [5, 12]. To our knowledge, CORA has never been studied specifically in our region. This study aims to describe demographic characteristics, clinical features, disease activity, functional disability and treatment of CORA at a tertiary hospital in Senegal (West Africa) and compare it with other studies.

## Methodology

This mixed cohort (retrospective and prospective) study was carried out at the rheumatology department of Aristide Le Dantec Hospital in Dakar (Senegal), from January 2020 to December 2022. This department is one of the two tertiary rheumatology departments in Dakar region. The population of Dakar metropolitan area is estimated at 4.04 million.

All patients who fulfilled the 2019 PRINTO preliminary criteria of positive RF-polyarthritis (arthritis for ≥ 6 weeks, two positive tests for RF at least three months apart or one positive test for anti-CCP) with an age of onset < 18 years were enrolled in the study [13]. In our practice, the rheumatoid factor test is usually combined with the anti-CCP test. Exclusion criteria were systemic arthritis, psoriasis arthritis, enthesitis related arthritis, acute rheumatic fever, and post-infectious arthritis. The following data were collected: - Age at symptom onset and at diagnosis (presentation), duration of symptoms (defined as the delay between symptom onset and diagnosis), and sex (female, male).

- Clinical features: morning stiffness joint pain, joint swelling, joint deformities (defined as ankylosed joints, or joints with standard deformities such as swan neck, boutonniere or Mallet deformity), extra-articular symptoms (Rheumatoid nodules, Interstitial lung disease), and family history of RA.
- Laboratory investigations: Haemoglobin (Anaemia if Hb < 12 g/dl), erythrocyte sedimentation rate (ESR, first hour; raised if > 20 mm/hour), C-reactive protein (CRP; positive if > 6 mg/l), rheumatoid factor (RF; positive if > 30 IU/ml by Waaler-Rose test), anti-cyclic citrullinated peptide (anti-CCP; positive if > 5 U/ml by chemiluminescence immunoassay test), antinuclear antibody (ANA; positive if > 1/100 by indirect immunofluorescence test).
- Radiography investigations: Plain radiographs of hands and wrists, erosive RA defined as the presence of erosions and/or other related abnormalities like joint space narrowing, carpitis, subluxations, ankylosis, and loss of mineralization.
- Disease activity was assessed by Disease Activity Score in 28 joints (DAS28-ESR and DAS28-CRP). High disease activity is defined as DAS28-ESR greater than 5.1. Moderate disease activity as a DAS28-ESR greater than 3.2 and less or equal to 5.1. Low disease activity as a DAS28-ESR less or equal to 3.2 and greater than 2.6. Clinical remission is defined as DAS28-ESR or CRP < 2.6.
- Functional disability at diagnosis was assessed using Health Assessment Questionnaire (HAQ) or if not possible by Childhood HAQ. The range of HAQ scores is between 0 and 3, where 0 represents no disability and 3 indicates complete disability.
- Treatment options were noted: conventional synthetic disease-modifying anti-rheumatic drugs: methotrexate (0.3–0.5 mg/kg/week), hydroxychloroquine (3–5 mg/kg/day), sulfasalazine (50 mg/kg/day). Glucocorticoid: oral prednisone (0.1–0.5 mg/kg/day) and intra-articular injections (Triamcinolone acetonide or Betamethasone). NSAIDs (naproxen, celecoxib).
- Follow-up: after initiation of treatment, we follow-up patients during six months. Disease activity was assessed after this period of therapy using the DAS28-CRP score.

Given the nature of this study, informed consent for participation was not required for retrospective data. However, for prospective data consent was obtained from the children’s parents. Confidentiality was ensured for all participants.

### Data analysis

Statistical analysis of data was done using Statistical Package for Social Sciences (SPSS) version 21.0 for Windows. Descriptive analysis was done and statistics were presented as numbers and percentages for categorical data and mean and standard deviation (SD) for continuous data. The Pearson correlation coefficient was used. P-values < 0.05 was considered statistically significant.

## Results

### Demographic and clinical features

**Fig. 1:**
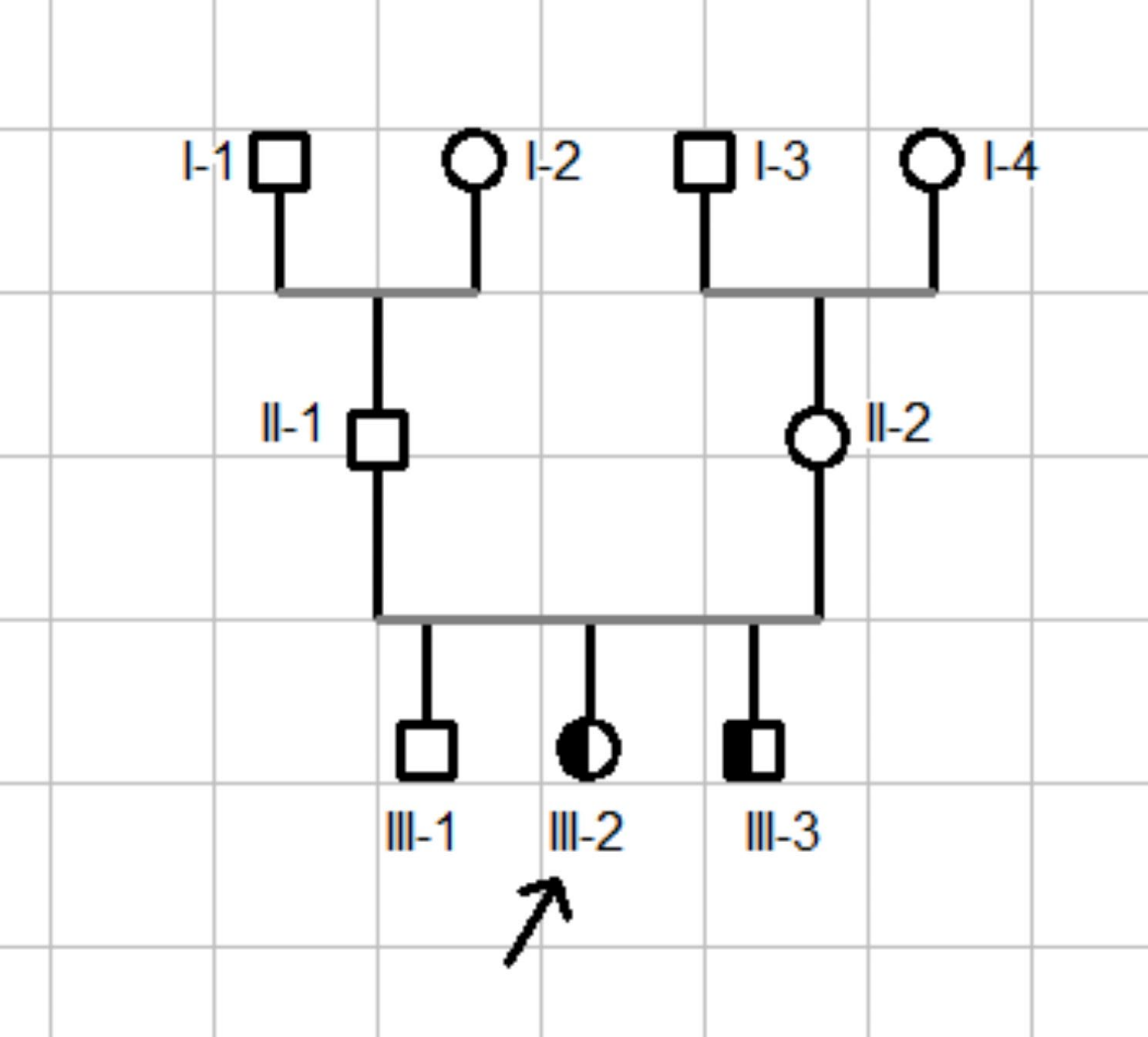
Familial Case of CORA; index case (III-2) 16 years old female with CORA with both positivity FR and Ac-CCP, his brother (III-3) 12 years old with CORA positive Ac-CCP/ negative RF (circles and squares indicate females and males respectively)

**Fig. 2:**
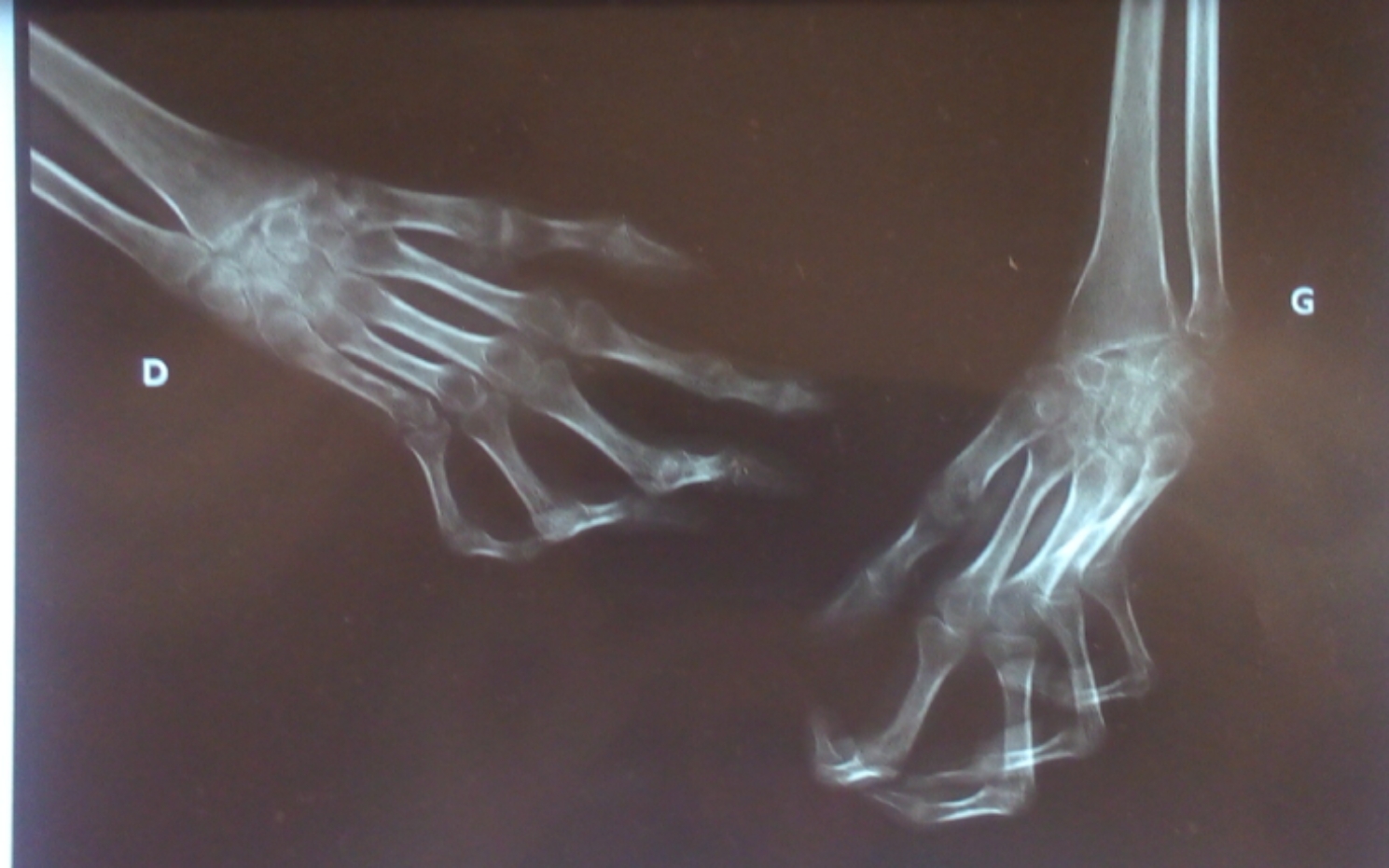
Joints deformities and ankyloses affecting both hands and wrists in an 18-year-old female with CORA started at 15 years old

A total of 29 patients were enrolled in the study. Eight children were excluded due to insufficient clinical data collection, leaving 21 patients with childhood-onset RA. Descriptive data for all patients are presented in Table 1. The female: male ratio was 6:1 (18 F:3 M). The mean age at symptom onset and at the time of diagnosis of CORA were 13 ± 3.0 years and 16.4 ± 4.2 years, respectively. The mean duration of symptoms was 3.5 ± 3.3 years. The main articular symptoms were joint pain in 21 (100%), followed by morning stiffness in 20 (95.2%), joint swelling in 15 (71.4%) patients, and joint deformities found in 12 (57.1%) patients. Extra-articular manifestations were represented by rheumatoid nodules in 5 (23.8%) patients and two patients with interstitial lung disease. Four patients had a family history of rheumatoid arthritis. Figure 1, illustrates a familial case of childhood-onset rheumatoid arthritis in this study. In terms of functional disability, the median CHAQ/HAQ score was 2.1 at diagnosis with a mean of 1.9 ± 0.92. Among the X-rays available, childhood-onset RA was erosive in 15 of 17 cases. The Fig. 2 shows joint deformities in a female with CORA.

**Table 1:** Demographic and clinical characteristics of 21 patients CORA at diagnosis

Variable	Number (%)	Mean (± SD)	Range
Sex			
Female	18 (85.7)		
Male	3 (14.3)		
Age (years)			
Age at symptom onset		13.04 (± 3.0)	6–17
Age at diagnosis (presentation)		16.4 (± 4.2)	7–25
Articular symptoms			
Morning stiffness	20 (95.2)		
Joint pain	21 (100)		
Joint swelling	15 (71.4)		
Joint deformities	12 (57.1)		
Extra-articular symptoms			
Rheumatoid nodules	5 (23.8)		
Interstitial lung disease	2 (9.5)		
Family history of RA	4 (19.04)		
CHAQ/HAQ score		1.89 ± 0.92	0.5-3

### Laboratory features and disease activity

The biological inflammatory syndrome was constant in our patients. ESR was accelerated in 90% of patients, with an average of 48.6 mm/h (Table 2). C-reactive protein was elevated in 85.7% of patients with a mean of 59.3 mg/l. Inflammatory anaemia was present in 13 cases (62%), the mean haemoglobin concentration was 11.6 (8.4–15.7) g/dl. Anti-CCP were positive among 18 of 20 (90%) patients, while 16/21 (76.2%) patients had positive RF. ANA was positive in 7 of 12 patients. Of the ANA-positive patients, one patient had juvenile Sjogren’s syndrome associated with rheumatoid arthritis, and the other patient had Rhupus syndrome.

In terms of disease activity, the mean DAS28-ESR was 5.7 ± 1.0 and 71.4% (15/21) of patients had a high active disease, while six patients (28.6%) had moderate disease activity. Functional disability was correlated with disease activity (r = 0.71, P < 0.01). In contrast, disease duration prior to treatment initiation was not correlated with disease activity (r= -0.15, P = 0.5).

### Therapeutic regime and follow-up

A wild majority of patients (19/21) (90.5%) were treated with methotrexate, while 17 (81%) patients had a combination of methotrexate and hydroxychloroquine. One patient had a combination of methotrexate and sulfasalazine. Oral prednisone was used in 17 (81%) cases while non-steroidal anti-inflammatory drugs (NSAIDs) were used in 4 cases (19%). Intra-articular joint steroid injections were performed in 7 (33.3%) patients.

In this cohort, among 21 patients included, four patients have been lost to follow-up. After six months of treatment, the average DAS-28 CRP was 2.90 with a median of 2.84 ± 0.93. Moderate disease activity occurred in 5/17 (29.4%) patients, while six (35.3%) presented low disease activity. Clinical remission was observed in 6/17 (35.3%) patients.

**Table 2:** Laboratory characteristics and disease activity of patients with CORA at diagnosis

Laboratory abnormalities	Total number of CORA cases tested	Number (%)
Anaemia (< 12 g/dl)	21	13 (62)
Raised ESR (ESR ≥ 20 mm/h)	20	18 (90)
Elevated CRP (CRP ≥ 6 mg/l)	19	18 (85.7)
Positive RF	21	16 (76.2)
Positive Anti-CCP antibody	20	18 (90)
Negative RF and Positive Anti-CCP	21	5 (23.8)
Both RF and anti-CCP positivity	20	13 (65)
Positive Anti-nuclear antibody	12	7 (58.3)
**Laboratory parameters**	**Mean (± SD)**	**Range**
Haemoglobin	11.6 (± 1.6)	8.4–15.7
ESR (mm/h)	48.6 (± 29.1)	10–110
CRP (mg/l)	59.3 (± 59.5)	0-192
**Disease activity score**	**Mean (± SD)**	**Range**
DAS28-ESR	5.7 (± 1.0)	4.0-7.2
DAS28-CRP	5.4 (± 1.1)	3.1–7.1

## Discussion

To our knowledge, this study is the first in sub-Saharan Africa to specifically describe childhood-onset rheumatoid arthritis. Previous studies have described CORA (RF-positive polyarthritis) in the context of juvenile idiopathic arthritis using ILAR criteria [14, 15]. The particularity of this current study is using 2019 PRINTO criteria for CORA, which includes research on Ac-CCP antibodies which are more specific than rheumatoid factors in rheumatoid arthritis [16, 17].

The ILAR criteria for RF-positive polyarthritis represent diagnosis limitations for children with Ac-CCP+/RF- polyarthritis because anti-CCP is not part of the diagnostic criteria. Since then, the inclusion of Ac-CCP positivity in the recent PRINTO provisional criteria for RF-positive polyarthritis would make it possible to include children with RF-/CCP + polyarthritis in this category.

The term “Childhood-onset rheumatoid arthritis” is more appropriate than “RF-positive polyarthritis JIA”, as it better defines the characteristics of the disease (positive anti-CCP and/or RF test, presence of erosions). This allows a better understanding of the disease nomenclature between adult rheumatologist and paediatric rheumatologist.

In this study, there was a female predominance (18 F:3 M) consistent with other studies in sub-Saharan Africa [14, 15]. Generally, RF-positive polyarticular JIA (CORA) is most common in adolescent girls (10:1, F/M) of all ethnic backgrounds [18]. The mean age of onset in our patients was 13 years, which was similar to the Indian study [19]. However, earlier average ages have been reported in South Africa (10 years) [14] and Zambia (11 years) [15]. In the Western literature, the most frequently reported age of onset of CORA was 11.4 years, which was almost similar to our series [20]. The mean age at diagnosis was 16.4 years in keeping with earlier studies from sub-Saharan Africa (Table 3) [14, 15, 21].

The long duration of symptoms averaging 3.5 years is linked to several factors which constitute barriers to early diagnosis and treatment in our context. We can suggest three possible explanations for that. Firstly, some patients initially favour the use of traditional medicines (phytotherapy), before consulting in the hospital at a later stage. Secondly, the unawareness of this disease in primary health care facilities, which sometimes diagnose it as rheumatic fever. Thirdly, is the scarcity of rheumatology specialists; in the absence of a pediatric rheumatology unit, the rheumatologist manages both adults and children.

Remarkably, approximately 19% of patients reported a family history of RA in this current study, supporting a major role for genetic factors. In Senegal, Dieye et al. [22] demonstrated that HLA-DR10 and HLA-DR3 were positively associated with adult-onset RA. Moreover, familial forms of rheumatoid arthritis were reported in 17 Senegalese multiplex families [23]. In Europe and the United states of America, Hinks et al. [11] showed that the genetic profile of CORA was similar to adult rheumatoid arthritis. However, no genetic studies have been carried out to confirm these results in Senegalese patients.

Among extra-articular manifestations, rheumatoid nodules were the most frequent extra-articular in our series (23.8%). This result was in agreement with the literature. Indeed, rheumatoid nodules founded in about 30% of children with RF-positive polyarticular JIA [18].

Interstitial lung disease (ILD) was the most common and severe manifestation of RA lung disease [24] occurring in 7.7-67% of patients [25]. In this current study, only two patients (9.5%) had ILD, this result was probably underestimated by the fact that we used only the chest X-ray as an investigation. Thus, high-resolution computed tomography imaging is more specific and sensitive for detecting ILD in rheumatoid arthritis patients [26, 27].

In our series, the highly disease activity score at diagnosis was probably related to the long duration of symptoms and diagnostic delays.

In our study, 90% of patients were positive for Anti-CCP antibodies, highlighting the key role of these autoantibodies in diagnosing Childhood-onset RA. Therefore, Rheumatoid Factor appears to be less effective than Ac-CCP (76.1% vs. 90%) in the diagnosis of this cohort. Indeed, five (23.8%) patients had negative rheumatoid factors, but positive anti-CCP, suggesting a systematic search for Ac-CCP in children with RF-negative polyarthritis, to avoid missing a childhood-onset RA. Among 13 of 20 patients had both RF and Ac-CCP positivity. This double positivity has been associated with more severe and more erosive diseases [28]. This explains the presence of joint deformities in this study.

Positive ANA frequency varies in the literature (7.6 − 75%) [19, 29–31]. Nishimura et al. [32] showed that the prevalence of ANA positivity in patients with advanced or prolonged rheumatoid arthritis was higher than in those with early stages or short durations.

Functional disability was much impaired in our patients, compared to the South African and Guinean studies (1 and 1.1 respectively) related to long duration of symptoms, and the presence of joint deformities.

Regarding drug therapy, we initially resorted to csDMARDs in response to a high level of disease activity in our patients. In Senegal, where resources are limited tumor necrosis factor alpha (TNFα) inhibitors are unavailable. We use a combination of methotrexate and hydroxychloroquine associated with glucocorticoids (oral, intra-articular injections) or NSAIDs.

**Table 3:** Comparative Data for Childhood-onset RA (RF-positive JIA)

Study	South Africa [14]	Zambia [15]	India [19]	Turkey [30]	Senegal
Number	11	9	28	13	21
Female: Male Ratio	NA	9:0	8:1	9:4	18:3
Age at onset	10	11	13	10.4	13
Age at presentation	15	16	NA	NA	16.4
ESR	42	53	NA	NA	48.6
Positive ANA %	NA	NA	27.3	7.6	58.3
CHAQ	1.0	NA	NA	NA	1.9

NA: Not available

Several limitations of our study should be noted. This cohort was mixed, with some retrospective data. The other extra-articular manifestations such as ocular and cardiac involvement were not studied. The disease activity at diagnosis was probably under-evaluated because, some patients initially received analgesic treatment (Paracetamol, NSAIDs, corticosteroids) in primary health before referral.

## Conclusion

In our study, childhood-onset rheumatoid arthritis affects most commonly adolescent 13 years old girls. It is characterized by high prevalence of Ac-CCP (90%). The disease was severe at diagnosis with high disease activity, joint deformations and significant functional disability. Treatment was based mainly on csDMARDs and corticosteroids. The data support research on Ac-CCP in children with RF-negative polyarthritis. However, further prospective studies are needed, including research into genetic factors, to obtain reliable clinical phenotypes of this disease, especially in sub-Saharan Africa.

## Data Availability

The datasets from this study are available on request from the corresponding author.

## Abbreviations

ANA: Anti-nuclear antibody
CHAQ: Childhood Health Assessment Questionnaire
CORA: Childhood-onset Rheumatoid Arthritis
CRP: C-reactive protein
csDMARDs: Conventional synthetic disease-modifying anti-rheumatic drugs
DAS: Disease activity score
ESR: Erythrocyte sedimentation rate
HAQ: Health Assessment Questionnaire
ILD: Interstitial lung disease
JIA: Juvenile idiopathic arthritis
PRINTO: Paediatric Rheumatology International Trials Organization
RA: Rheumatoid arthritis
RF: Rheumatoid factor
